# DIET AND NUTRITION: The Artificial Food Dye Blues

**DOI:** 10.1289/ehp.118-a428

**Published:** 2010-10

**Authors:** Carol Potera

**Affiliations:** **Carol Potera**, based in Montana, has written for *EHP* since 1996. She also writes for *Microbe*, *Genetic Engineering News*, and the *American Journal of Nursing*

In 2008 the Center for Science in the Public Interest (CSPI) in Washington, DC, petitioned the Food and Drug Administration (FDA) to ban artificial food dyes because of their connection to behavioral problems in children.^1^ Two years later a new CSPI report, *Food Dyes: A Rainbow of Risks*, further concludes that the nine artificial dyes approved in the United States likely are carcinogenic, cause hypersensitivity reactions and behavioral problems, or are inadequately tested.^2^

Artificial dyes derived from petroleum are found in thousands of foods.^3^ In particular breakfast cereals, candy, snacks, beverages, vitamins, and other products aimed at children are colored with dyes. Even some fresh oranges are dipped in dye to brighten them and provide uniform color, says Michael Jacobson, executive director at CSPI.

According to the International Association of Color Manufacturers, a trade association for food dye makers and users, artificial color additives enhance and correct natural colors and “provide a colorful identity to foods that would otherwise be virtually colorless,” as well as compensating for natural color loss during storage and providing a way to quickly identify pharmaceuticals and dietary supplements.^4^ Food dye consumption per person has increased fivefold in the United States since 1955, with three dyes—Red 40, Yellow 5, and Yellow 6—accounting for 90% of the dyes used in foods.^2^

For its report CSPI reviewed published studies and “found some surprises,” says Jacobson. For example, most chemical carcinogenicity studies use relatively small numbers of animals, do not include *in utero* exposures, and last two years, the rodent equivalent of about 65 human years.^5^ Because cancers may not show up until a rodent’s third year of life, corresponding to the time when cancers also are more likely to appear in humans, the two-year time frame for standard bioassays may reduce the likelihood a carcinogenic chemical will be identified, says James Huff, associate director for chemical carcinogenesis at the National Institute of Environmental Health Sciences.

Red 40, Yellow 5, and Yellow 6 contain benzidene, a human and animal carcinogen permitted in low, presumably safe levels in dyes.^2^ The FDA calculated in 1985 that ingestion of free benzidine raises the cancer risk to just under the “concern” threshold (1 cancer in 1 million people).^6^ Bound benzidene also has been detected in dyes in much greater amounts than free benzidene,^7^,^8^ but routine FDA tests measure only free contaminants, overlooking the bound moiety.^2^ Intestinal enzymes release bound benzidene, “so we could be exposed to vastly greater amounts of carcinogens than FDA’s routine tests indicate,” says Jacobson—especially considering today’s children are exposed to multiple dyes and flavoring agents and other added chemicals in foods.^9^

FDA policy is not to comment on topics that are currently under review. This includes CSPI’s open 2008 petition, whose docket of evidence now includes the new report. Ira R. Allen of the FDA Office of Public Affairs did say, “We appreciate the report from CSPI and are reviewing it. We take our commitment to protecting children seriously.” In a statement released after the publication of *A Rainbow of Risks*, the International Association of Color Manufacturers highlighted its adherence to current FDA protocols, noting, “The FDA has repeatedly stated that these colors are safe based on the available safety data.”^4^

Food manufacturers still use plant-based colorings in some countries. For example, in the United Kingdom Fanta orange soda is colored with pumpkin and carrot extracts while the U.S. version uses Red 40 and Yellow 6. McDonald’s strawberry sundaes are colored only with strawberries in Britain, but Red 40 is used in the United States. With many U.S. consumers desiring fewer synthetic additives, “companies may be better off switching to [plant-based colors],” Jacobson says.

“Natural alternatives may present less of a risk, but I still would like to see their toxic potential assayed before we give them to kids,” says Bernard Weiss, a professor of environmental medicine at the University of Rochester. Weiss argued 30 years ago there was evidence linking artificial food dyes to behavioral problems in children.^10^ Yet the FDA still does not require manufacturers to test dyes for developmental neurotoxicity. “Their inaction amounts to approval of an ongoing experiment with children,” Weiss says.

Meanwhile, in Europe, as of July 2010 most foods that contain artificial dyes must carry labels warning they may cause hyperactivity in children.^11^ Jacobson says, “This warning may be the death knell for [artificial] food dyes in Europe, especially for foods commonly eaten by children.”

## Figures and Tables

**Figure:**
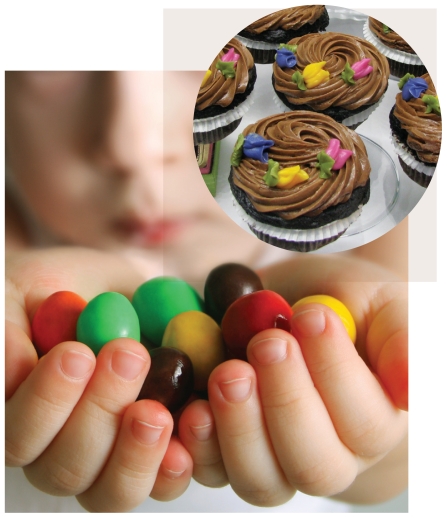
Artificial dyes are not the only way to create brightly colored foods; many countries use vegetable-based dyes (see sample wares in the inset) to achieve the same effect.

## References

[1] CSPI Petition to Ban the Use of Yellow 5 and Other Food Dyes, in the Interim to Require a Warning on Foods Containing these Dyes, to Correct the Information the Food and Drug Administration Gives to Consumers On the Impact of These Dyes on the Behavior of Some Children, and to Require Neurotoxicity Testing of New Food Additives and Food Colors.

[2] CSPI (2010). Food Dyes: A Rainbow of Risks.

[b3-ehp-118-a428] 3See list at http://tinyurl.com/287p2xz [accessed 15 September 2010].

[4] IACM (2010). International Association of Color Manufacturers Reaffirms Safety of Food Dyes [press release].

[5] Huff J (2008). Environ Health Perspect.

[6] FD&C Yellow No. 5; Agency: Food and Drug Administration (1985). Action: Final rule, removal of stay. Fed Reg.

[7] Peiperl MD (1995). Food Chem Toxicol.

[8] Lancaster FE, Lawrence JF (1999). Food Additives Contaminants Part A.

[b9-ehp-118-a428] 9In August 2010, the U.S. Environmental Protection Agency announced it planned to initiate rulemaking to regulate 48 benzidene-based dyes under the Toxic Substances Control Act. The act has authority for nonfood uses of these dyes in products such as textiles and inks. For more information see http://tinyurl.com/25ta7pg [accessed 15 Sep 2010].

[10] Weiss B (2000). Environ Health Perspect.

[11] Food Standards Agency (2010). Compulsory Warnings on Colours in Food and Drink [press release].

